# Decoding Emergency Department Dissatisfaction: Factors Associated with Patient Complaints

**DOI:** 10.5811/westjem.48866

**Published:** 2026-02-22

**Authors:** Mitchell Blenden, Rohit B. Sangal, Craig Rothenberg, Wendy W. Sun, Kwame Tuffuor, Suresh K. Pavuluri, Reinier Van Tonder, Sharon Chekijian, Eleanor Reid, Vivek Parwani

**Affiliations:** Yale University School of Medicine, Department of Emergency Medicine, New Haven, Connecticut

## Abstract

**Introduction:**

Patient experience has important implications for hospitals and patient care including its ties to reputation, reimbursement, and clinical outcomes. Despite its importance, little is known about how operational factors in the emergency department (ED) impact formal complaints. In this study we aimed to identify encounter-level operational characteristics associated with the risk of formal patient complaints.

**Methods:**

We conducted a retrospective matched-cohort study of ED encounters between October 2023–December 2024 at three EDs affiliated with a large academic health system. Each complaint case was matched to three non-complaint cases (3:1 matching) based on age, sex, race/ethnicity, acuity score, and chief complaint. We used logistic regression to assess the associations between operational factors and the likelihood of submitting a formal complaint. A Bonferroni correction was applied for multiple comparisons with statistical significance set at P < .005.

**Results:**

Of 246,983 ED visits, 476 (0.19%) formal complaints were submitted. These were matched with 1,428 non-complaint cases. Baseline characteristics, which included age, sex, race/ethnicity, primary insurance, and chief complaint, did not differ, by design, between groups. Analysis revealed that ED length of stay ≥ 12 hours (odds ratio OR 3.12; 95% CI, 2.34–4.18) and an average of more than one ED visit per month (2.00; 1.45–2.73) were significantly associated with increased odds of filing a complaint. In contrast, any imaging performed during the visit (0.43; 0.35–0.54), hospital admission (0.72; 0.57–0.90), and presenting to the ED during a high-volume time (0.47; 0.33–0.67) were significantly associated with decreased odds of filing a complaint.

**Conclusion:**

Length of stay > 12 hours and frequent ED visits were associated with a significantly increased complaint risk. Any form of diagnostic imaging, admission to the hospital, and presenting to the ED during a high-volume period were associated with fewer complaints. These findings offer ED and hospital leadership insights on the patient experience and highlight that improving capacity constraints for all patients can have downstream benefits for those who submit formal complaints.

## INTRODUCTION

Patient experience is important for hospital performance and is tied to reimbursement, public perception, and clinical outcomes. Programs such as Medicare’s Value-Based Purchasing initiative and private payor contracts link financial incentives to patient experience metrics, underscoring their importance both operationally and financially.^1^,^2^ Improved patient satisfaction has also been linked to better clinical outcomes.^1^,^3^ In contrast, dissatisfied patients are more likely to leave negative reviews, file complaints, or pursue legal action, further impacting hospital operations and reputation.^4^,^5^

Given that many hospital admissions come through the emergency department (ED),^6^–^8^ understanding which operational aspects of ED care are most strongly associated with dissatisfaction is essential for improving patient experience, optimizing ED workflows, and mitigating financial risk. Emergency departments are increasingly strained by high patient volumes, prolonged boarding times, and frequent use of hallway beds, all of which can negatively affect environment and set the tone for a poor patient experience.^9^,^10^ Several prior qualitative studies of ED complaints describe recurring themes including wait times, respect, and communication.^11^,^12^ These studies, however, rarely link themes to encounter-level operational measures. Our study addresses this gap by quantifying which specific operational-level characteristics of an ED visit are associated with the submission of a formal complaint.

We focus on formal written complaints rather than general satisfaction as these are high-impact events that trigger institutional review and may carry regulatory reporting obligations. Unlike deidentified satisfaction surveys, formal complaints are identifiable and auditable, allowing linkage to encounter-level operations and targeted quality improvement interventions. In this study we aimed to identify encounter-level drivers of patient dissatisfaction by examining which ED characteristics are most associated with formal complaints.

## METHODS

### Study Design and Setting

We conducted a retrospective matched cohort study of ED patients within a single, academic health system in the Northeast United States with 200,000 annual patient encounters. The study was conducted between October 2023–December 2024.

### Data Collection

We extracted data from the enterprise data warehouse (Epic Systems Corporation, Verona, WI) on all ED visits during the study period, including operational variables and complaint data. Operational variables included the following: ED length of stay; time from arrival to being seen by a clinician; time from arrival to being placed in a room; disposition status (admission or discharge); boarding time (duration from admission order to ED departure with a threshold of ≥ 4 hours); hallway bed placement; frequent ED utilization (more than one visit per month); return visits within 72 hours: arrival time (day time, 7 am–6 pm; or nighttime 6 pm–7 am); whether any imaging (radiograph, computed tomography, ultrasound or magnetic resonance imaging) was performed; the proportion of each patient’s stay that overlapped with high-volume hours (top 30% of occupancy for the site); and the patient’s primary insurance status.

**Population Health Research Capsule**

What do we already know about this issue?

*Patient complaints reflect gaps in patient experience, but the specific (ED) operational factors that drive complaints are unclear*.

What was the research question?

*What operational factors are associated with ED complaints?*

What was the major finding of the study?

*Length of stay ≥ 12 hours was associated with 3-fold higher complaint odds (odds ratio 3.12, 95% CI 2.34–4.18; P < .001)*.

How does this improve population health?

*Identifying which ED operational factors drive complaints helps target system improvements that reduce dissatisfaction, improve care experiences, and supports equity*.

Complaints were extracted as formal complaints made through the patient relations department and formally logged in the tracking system (Situational Awareness for Emergency Response (SAFER), Press Ganey). Visits associated with complaints were considered “complaint cases.” Non-complaint visits were also drawn from the same time frame and used for matching as controls.

### Statistical Analysis

Each complaint case was matched to three control visits without a complaint (3:1 matching ratio). Matching was based on age, sex, race/ethnicity, acuity, and chief complaint. Chief complaints were characterized based on a previously validated classification scheme.^13^ To avoid self-matching, patients with a complaint visit were excluded from the control pool if they had another non-complaint ED visit. We conducted an unadjusted logistic regression to evaluate the association between each operational variable and the likelihood of a patient filing a complaint. A Bonferroni correction was applied to adjust for multiple comparisons, with a statistical significance set at *P* < .005. All analyses were conducted using R v4.2.2 (R Foundation for Statistical Computing, Vienna, Austria).

## RESULTS

### Sample Characteristics

A total of 246,983 patients were seen during the study period, of whom 476 (0.19%) submitted a complaint. These 476 complaints were matched to 1,428 non-complaint cases (Table 1). Baseline demographics between groups were similar, with no significant differences in age, sex, race/ethnicity, or chief complaint (Supplemental Table 1). Full pre-matching baseline characteristics are listed in Supplemental Table 2.

**Figure 1:**
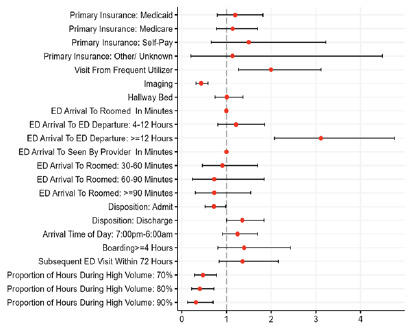
Odds ratios (OR) and 95% confidence intervals in a study of factors associated with patient-submitted complaints in the emergency department. The ORs were developed from a multivariate logistic regression of 476 complaints matched to 1,428 non-complaints. The vertical line denotes an OR of 1.

Regression analysis identified several operational factors that were significantly associated with the likelihood of a formal complaint. Patients with an ED LOS ≥ 12 hours had a 3.12 (2.34–4.18) increased odds of filing a complaint and frequent ED users had a 2.00 (1.45–2.73) increased odds of filing a complaint. Factors associated with decreased odds of filing a complaint included undergoing imaging during an ED visit (odds ratio [OR] 0.43; 95% CI, 0.35–0.54), admission to the hospital (0.72; 0.57–0.90) and greater exposure to high-volume hours during the ED (0.47; 0.33–0.67]). Boarding (> 4 hours) was not statistically significant. These findings are summarized in Figure 1 and detailed in Supplemental Table 3.

## DISCUSSION

In this study we examined the relationship between ED operational factors and the likelihood of patients to submit formal complaints. Over our study period, there were almost 247,000 ED visits from which we identified 476 complaints. We found that prolonged LOS in the ED (≥ 12 hours) and frequent ED users (> 1 visit per month) significantly increased the likelihood of a complaint, while admission to the hospital, diagnostic imaging, and arrival during high-volume periods were associated with a decreased likelihood of filing a complaint.

This study adds to the existing body of literature on the patient experience by focusing on formal complaints rather than survey-based satisfaction scores, which has been the primary focus of much prior ED satisfaction research. While earlier studies have linked ED operational factors to the patient experience, they typically relied on survey data.^14^–^17^ In contrast, ours is the first study to our knowledge to use a matched cohort design and patient-level operational metrics to identify factors associated with formally submitted complaints, providing a novel, quantifiable, and actionable approach to patient dissatisfaction in the ED.

Prolonged ED stays, particularly those > 12 hours, were among the factors most strongly associated with the filing of a formal complaint. This is consistent with prior research linking operational delays, particularly longer wait times and longer ED LOS, to worse patient experience measures.^18^–^20^ These findings support constant administrative efforts to streamline throughput. Since time spent boarding for an inpatient bed beyond four hours was not independently associated with complaints, the prolonged ED stays we captured (> 12 hours) likely reflect a different subset of patients, ie, those still awaiting final disposition. Often these patients are in ED observation status awaiting specialty consultations or advanced imaging. These encounters involve multiple hand-offs and infrequent updates, which may heighten frustration and prompt complaints.^21^

Frequent ED users were also more likely to file a complaint. Frequent users may have more complex or unmet needs, potentially predisposing them to file complaints. Their repeated visits may stem from a variety of reasons, including chronic conditions, care fragmentation, or social issues, all of which can lead to increased dissatisfaction. Targeted care coordination, enhanced linkages to outpatient resources, and improved communication may help reduce dissatisfaction in this group.^22^

Conversely, undergoing any form of imaging during an ED visit, was associated with a significantly lower likelihood of submitting a complaint. This may reflect the reassuring effect of diagnostic testing, as patients may perceive testing as indicative of their concerns being taken seriously; and when they are brought for an imaging study they may feel that this is an escalation of evaluation. Imaging also provides a tangible product in the form of a result that can reduce feelings of diagnostic uncertainty. Alternative explanations merit consideration, however, as those who do not undergo imaging may disproportionally present with less straightforward issues that are common among repeat presenters (ie, systemic frustrations or social needs) or may include encounters in which a diagnosis was missed due to imaging not being performed. As our variable included any imaging regardless of indication or result, this association likely serves as a proxy for evaluation thoroughness.

Although prolonged ED LOS was associated with higher odds of a complaint being filed and imaging was associated with lower odds these findings are not necessarily contradictory. Prolonged stays often reflect downstream operational factors rather than the diagnostic workup itself that likely drive dissatisfaction. In encounters with both imaging and prolonged stays these operational factors plausibly outweigh reassurance from testing. Future work should examine whether this association persists within similar chief complaints and complaint types and explore interactions between LOS and imaging.

Along these same lines, hospital admission was protective against a complaint, further supporting the role of perceived thoroughness in shaping patient experience. Similarly, being in the ED during a high-volume period was also associated with fewer complaints. This suggests that when patients witness a busy department, their expectations adjust accordingly, and they are more tolerant of delays and longer stays in the ED. This is consistent with attribution theory, which posits that individuals are less likely to blame service providers when delays appear uncontrollable^23^ and with prior ED research demonstrating that satisfaction hinges on perception.^24^ With this in mind, the “proportion of hours during high volume” variable measures the proportion of each encounter overlapping with peak census. Very long ED stays inevitably span both peak and off-peak periods, which lowers this proportion and may partly explain the observed protective association with high-volume periods.

Our findings offer several actionable insights for ED leadership. Interventions aimed at improving patient flow and addressing factors driving frequent ED utilization may help reduce dissatisfaction and its downstream consequences. As patient experience increasingly influences reimbursement, institutional reputation, and quality metrics, systematically identifying and addressing operational drivers of dissatisfaction remains a critical component of ED quality improvement initiatives.

## LIMITATIONS

These findings should be interpreted within the context of several limitations. First, this study only captured those who formally submitted complaints, which does not capture all patients who were dissatisfied and could under-represent or exclude certain populations. Second, complaint narrative text was not available for research use. Consequently, we could quantify associations between operational factors and the occurrence of a formal complaint, but we could not determine whether the content of the complaint explicitly pertained to those factors. Additionally, this study was limited to a single health system, which may affect generalizability. Finally, although we performed multivariable regression after matching, residual confounding cannot be excluded.

## CONCLUSION

Operational factors significantly shape the ED experience. Prolonged stays and frequent users were associated with an increased risk of formal complaints, while imaging, admission to the hospital, and exposure to high-volume times were protective against complaints. These findings highlight opportunities for quality improvement and to improve the patient experience with efforts focused on throughput and address care issues for frequent ED users. Future work should test targeted interventions and explore the protective associations observed with imaging, hospital admission, and high-volume periods.

## Supplementary Information







## Figures and Tables

**Table 1:** Baseline demographic and operational characteristics of complaint and non-complaint emergency department visits after matching in a study of patient-submitted complaints at three hospitals across a large academic health system.

After matching		

Characteristic	Complaint, n = 4761	No complaint, n = 14,281
Age	57 (40, 71)	57 (42, 71)
Sex		
Female	240 (50%)	726 (51%)
Male	236 (50%)	702 (49%)
Race ethnicity		
White non-Hispanic	229 (48%)	708 (50%)
Black non-Hispanic	155 (33%)	487 (34%)
Hispanic	63 (13%)	156 (11%)
Other/unknown	29 (6.1%)	77 (5.4%)
Primary insurance		
Commercial	101 (21%)	344 (24%)
Medicaid	151 (32%)	430 (30%)
Medicare	197 (41%)	589 (41%)
Other/unknown	27 (5.7%)	65 (4.6%)
ED disposition		
Admit	144 (30%)	537 (38%)
AMA	11 (2.3%)	21 (1.5%)
Discharge	293 (62%)	774 (54%)
Eloped	14 (2.9%)	19 (1.3%)
Other	14 (2.9%)	77 (5.4%)
Subsequent ED visit within 72 hours	57 (12%)	130 (9.1%)
Visit from frequent user	72 (15%)	117 (8.2%)
ED hallway flag	182 (38%)	543 (38%)
Time interval, ED arrival to ED departure in minutes bins		
< 4 hours	83 (17%)	404 (28%)
4–12 hours	168 (35%)	674 (47%)
≥ 12 hours	224 (47%)	350 (25%)
Boarding		
< 4 hours	61 (47%)	293 (55%)
≥ 4 hours	70 (53%)	241 (45%)
Imaging	243 (51%)	1,009 (71%)
Hours during high volume		
70 th percentile	11 (2, 30)	8 (2, 17)
80 th percentile	6 (1, 19)	4 (0, 12)
90 th percentile	2 (0, 10)	1 (0, 6)
Proportion of hours during high volume		
70	0.29 (0.11, 0.48)	0.33 (0.09, 0.63)
80	0.16 (0.02, 0.33)	0.17 (0.00, 0.46)
90	0.04 (0.00, 0.18)	0.05 (0.00, 0.22)

1 Median (interquartile range; n (%).

Definition of thresholds: 70th/80th/90th percentile refers to hours when real-time ED census exceeded the corresponding percentile of all hourly censuses during the study period. “Proportion of hours during high volume” refers to the fraction of each patient’s ED stay occurring during those high-volume times.

*AMA*, against medical advice; *ED*, emergency department.
