# Supplementary material for: Decoding Emergency Department Dissatisfaction: Factors Associated with Patient Complaints
Source: West J Emerg Med. 2026 Feb 22;27(2):244–9. doi: 10.5811/westjem.48866 (PMC13016072; doi:10.5811/westjem.48866)
Supplement: Supplementary file 1 [file wjem-27-244-s001.docx]

| **Supplemental Table 1.** Baseline demographics and operational characteristics after matching in a retrospective study of emergency department (ED) complaints.  After Matching |
| --- |
| Full Matching Data   \| After Matching \|  \|  \| \| --- \| --- \| --- \| \|  \|  \|  \| \|  \|  \|  \| \| **Characteristic** \| **Complaint**, N = 476*1* \| **No Complaint**, N = 1,428*1* \| \| Age \| 57 (40, 71) \| 57 (42, 71) \| \| Sex \|  \|  \| \| Female \| 240 (50%) \| 726 (51%) \| \| Male \| 236 (50%) \| 702 (49%) \| \| Race ethnicity \|  \|  \| \| White Non-Hispanic \| 229 (48%) \| 708 (50%) \| \| Black Non-Hispanic \| 155 (33%) \| 487 (34%) \| \| Hispanic \| 63 (13%) \| 156 (11%) \| \| Other/ Unknown \| 29 (6.1%) \| 77 (5.4%) \| \| Primary insurance \|  \|  \| \| Commercial \| 101 (21%) \| 344 (24%) \| \| Medicaid \| 151 (32%) \| 430 (30%) \| \| Medicare \| 197 (41%) \| 589 (41%) \| \| Self-Pay \| 22 (4.6%) \| 50 (3.5%) \| \| Other/ Unknown \| 5 (1.1%) \| 15 (1.1%) \| \| ED disposition \|  \|  \| \| Admit \| 144 (30%) \| 537 (38%) \| \| AMA \| 11 (2.3%) \| 21 (1.5%) \| \| Discharge \| 293 (62%) \| 774 (54%) \| \| Eloped \| 14 (2.9%) \| 19 (1.3%) \| \| Expired \| 9 (1.9%) \| 5 (0.4%) \| \| LWBS after Triage \| 4 (0.8%) \| 20 (1.4%) \| \| Observation \| 1 (0.2%) \| 40 (2.8%) \| \| Send to L&D \| 0 (0%) \| 4 (0.3%) \| \| Send to OR/Procedure \| 0 (0%) \| 2 (0.1%) \| \| Transfer to Another Facility \| 0 (0%) \| 6 (0.4%) \| \| Subsequent ED Visit within 72 hours \| 57 (12%) \| 130 (9.1%) \| \| ED arrival to ED departure in minutes \| 678 (320, 1,536) \| 373 (226, 705) \| \| Unknown \| 1 \| 0 \| \| ED arrival to seen by clinician in minutes \| 19 (9, 39) \| 18 (9, 42) \| \| Unknown \| 5 \| 25 \| \| ED arrival to roomed in minutes \| 6 (3, 13) \| 6 (4, 15) \| \| Unknown \| 4 \| 21 \| \| Admit dispo selected to ED departure in minutes \| 296 (182, 427) \| 224 (148, 354) \| \| Unknown \| 280 \| 819 \| \| Visit from frequent utilizer \| 72 (15%) \| 117 (8.2%) \| \| ED hallway flag Yn \| 182 (38%) \| 543 (38%) \| \| Time in hallway bed \| 266 (130, 483) \| 253 (136, 413) \| \| Unknown \| 294 \| 885 \| \| Arrival time of day \|  \|  \| \| 7:00am-6:00pm \| 292 (61%) \| 949 (66%) \| \| 7:00pm-6:00am \| 184 (39%) \| 479 (34%) \| \| Time interval ED arrival to ED departure in minutes bins \|  \|  \| \| <4 Hours \| 83 (17%) \| 404 (28%) \| \| 4-12 Hours \| 168 (35%) \| 674 (47%) \| \| >=12 Hours \| 224 (47%) \| 350 (25%) \| \| Unknown \| 1 \| 0 \| \| Time interval ED arrival to seen by clinician in minutes bins \|  \|  \| \| <30 Minutes \| 311 (66%) \| 939 (67%) \| \| 30-60 Minutes \| 91 (19%) \| 221 (16%) \| \| 60-90 Minutes \| 30 (6.4%) \| 125 (8.9%) \| \| >=90 Minutes \| 39 (8.3%) \| 118 (8.4%) \| \| Unknown \| 5 \| 25 \| \| Time interval ED arrival to roomed in minutes bins \|  \|  \| \| <30 Minutes \| 420 (89%) \| 1,220 (87%) \| \| 30-60 Minutes \| 26 (5.5%) \| 83 (5.9%) \| \| 60-90 Minutes \| 10 (2.1%) \| 40 (2.8%) \| \| >=90 Minutes \| 16 (3.4%) \| 64 (4.5%) \| \| Unknown \| 4 \| 21 \| \| Time interval admit dispo selected to ED departure in minutes bins \|  \|  \| \| <3 Hours \| 48 (24%) \| 211 (35%) \| \| 3-6 Hours \| 83 (42%) \| 253 (42%) \| \| 6-12 Hours \| 42 (21%) \| 103 (17%) \| \| >=12 Hours \| 23 (12%) \| 42 (6.9%) \| \| Unknown \| 280 \| 819 \| \| Boarding \|  \|  \| \| <4 Hours \| 61 (47%) \| 293 (55%) \| \| >=4 Hours \| 70 (53%) \| 241 (45%) \| \| Unknown \| 345 \| 894 \| \| Imaging \| 243 (51%) \| 1,009 (71%) \| \| Hours during high volume 70 \| 11 (2, 30) \| 8 (2, 17) \| \| Unknown \| 1 \| 0 \| \| Hours during high volume 80 \| 6 (1, 19) \| 4 (0, 12) \| \| Unknown \| 1 \| 0 \| \| Hours during high volume 90 \| 2 (0, 10) \| 1 (0, 6) \| \| Unknown \| 1 \| 0 \| \| Proportion of hours during high volume 70 \| 0.29 (0.11, 0.48) \| 0.33 (0.09, 0.63) \| \| Unknown \| 1 \| 0 \| \| Proportion of hours during high volume 80 \| 0.16 (0.02, 0.33) \| 0.17 (0.00, 0.46) \| \| Unknown \| 1 \| 0 \| \| Proportion of hours during high volume 90 \| 0.04 (0.00, 0.18) \| 0.05 (0.00, 0.22) \| \| Unknown \| 1 \| 0 \| \| \| *1* Median (IQR); n (%) \|  \| \| --- \| --- \| \| \| \|   *AMA*, against medical advice; *LWBS*, Left without being seen; *L&D*, Labor and delivery; *OR*, operating room; *IQR*, interquartile range |
