# Supplementary material for: Decoding Emergency Department Dissatisfaction: Factors Associated with Patient Complaints
Source: West J Emerg Med. 2026 Feb 22;27(2):244–9. doi: 10.5811/westjem.48866 (PMC13016072; doi:10.5811/westjem.48866)
Supplement: Supplementary file 2 [file wjem-27-244-s002.docx]

| Full Data Before Matching (Supplemental Table 2) | | |
| --- | --- | --- |
| **Supplemental Table 2.** Baseline demographics and operational characteristics before matching in a retrospective study of emergency department (ED) complaints. |  |  |
|  |  |  |
|  |  |  |
| **Characteristic** | **Complaint**, N = 476*1* | **No Complaint**, N = 246,507*1* |
| Age | 57 (40, 71) | 51 (33, 67) |
| Sex |  |  |
| Female | 240 (50%) | 132,068 (54%) |
| Male | 236 (50%) | 114,439 (46%) |
| Race ethnicity |  |  |
| White Non-Hispanic | 229 (48%) | 112,517 (46%) |
| Black Non-Hispanic | 155 (33%) | 64,156 (26%) |
| Hispanic | 63 (13%) | 53,678 (22%) |
| Other/ Unknown | 29 (6.1%) | 16,156 (6.6%) |
| Primary insurance |  |  |
| Commercial | 101 (21%) | 62,242 (25%) |
| Medicaid | 151 (32%) | 84,466 (34%) |
| Medicare | 197 (41%) | 81,429 (33%) |
| Self-Pay | 22 (4.6%) | 16,746 (6.8%) |
| Other/ Unknown | 5 (1.1%) | 1,624 (0.7%) |
| ED disposition |  |  |
| Admit | 144 (30%) | 59,069 (24%) |
| AMA | 11 (2.3%) | 2,103 (0.9%) |
| Discharge | 293 (62%) | 174,936 (71%) |
| Eloped | 14 (2.9%) | 2,773 (1.1%) |
| Expected cancelled by patient | 0 (0%) | 1 (<0.1%) |
| Expired | 9 (1.9%) | 261 (0.1%) |
| LWBS after Triage | 4 (0.8%) | 4,351 (1.8%) |
| LWBS before Triage | 0 (0%) | 3 (<0.1%) |
| No show | 0 (0%) | 1 (<0.1%) |
| Observation | 1 (0.2%) | 1,715 (0.7%) |
| Send to L&D | 0 (0%) | 338 (0.1%) |
| Send to OR/Procedure | 0 (0%) | 160 (<0.1%) |
| Transfer to Another Facility | 0 (0%) | 796 (0.3%) |
| Subsequent ED Visit Within 72 Hours | 57 (12%) | 23,100 (9.4%) |
| ED arrival to ED departure in minutes | 678 (320, 1,536) | 294 (177, 500) |
| Unknown | 1 | 2 |
| ED arrival to seen by clinician in minutes | 19 (9, 39) | 24 (11, 52) |
| Unknown | 5 | 4,675 |
| ED arrival to roomed in minutes | 6 (3, 13) | 9 (5, 24) |
| Unknown | 4 | 3,782 |
| Admit dispo selected to ED departure in minutes | 296 (182, 427) | 242 (154, 365) |
| Unknown | 280 | 181,133 |
| Visit from frequent utilizer | 72 (15%) | 20,922 (8.5%) |
| ED hallway flag Yn | 182 (38%) | 77,212 (31%) |
| Time in hallway bed | 266 (130, 483) | 228 (125, 364) |
| Unknown | 294 | 169,295 |
| Arrival time of day |  |  |
| 7:00am-6:00pm | 292 (61%) | 165,495 (67%) |
| 7:00pm-6:00am | 184 (39%) | 81,012 (33%) |
| Time interval ED arrival to ED departure in minutes bins |  |  |
| <4 Hours | 83 (17%) | 96,282 (39%) |
| 4-12 Hours | 168 (35%) | 110,900 (45%) |
| >=12 Hours | 224 (47%) | 39,323 (16%) |
| Unknown | 1 | 2 |
| Time interval ED arrival to seen by clinician in minutes bins |  |  |
| <30 Minutes | 311 (66%) | 139,696 (58%) |
| 30-60 Minutes | 91 (19%) | 50,795 (21%) |
| 60-90 Minutes | 30 (6.4%) | 22,031 (9.1%) |
| >=90 Minutes | 39 (8.3%) | 29,310 (12%) |
| Unknown | 5 | 4,675 |
| Time interval ED arrival to roomed in minutes bins |  |  |
| <30 Minutes | 420 (89%) | 192,056 (79%) |
| 30-60 Minutes | 26 (5.5%) | 24,041 (9.9%) |
| 60-90 Minutes | 10 (2.1%) | 9,871 (4.1%) |
| >=90 Minutes | 16 (3.4%) | 16,757 (6.9%) |
| Unknown | 4 | 3,782 |
| Time interval admit dispo selected to ED departure in minutes bins |  |  |
| <3 Hours | 48 (24%) | 21,404 (33%) |
| 3-6 Hours | 83 (42%) | 27,088 (41%) |
| 6-12 Hours | 42 (21%) | 12,766 (20%) |
| >=12 Hours | 23 (12%) | 4,116 (6.3%) |
| Unknown | 280 | 181,133 |
| Boarding |  |  |
| <4 Hours | 61 (47%) | 29,573 (52%) |
| >=4 Hours | 70 (53%) | 26,803 (48%) |
| Unknown | 345 | 190,131 |
| Imaging | 243 (51%) | 150,937 (61%) |
| \| *1* Median (IQR); n (%) \|  \| \| --- \| --- \| |  |  |

*AMA*, against medical advice; *LWBS*, Left without being seen; *L&D*, Labor and delivery; *OR*, operating room; *IQR*, interquartile range
