# Supplementary material for: Decoding Emergency Department Dissatisfaction: Factors Associated with Patient Complaints
Source: West J Emerg Med. 2026 Feb 22;27(2):244–9. doi: 10.5811/westjem.48866 (PMC13016072; doi:10.5811/westjem.48866)
Supplement: Supplementary file 3 [file wjem-27-244-s003.docx]

Regression Data

**Supplemental Table 3.** Logistic Regression results for each operational factor associated with emergency department (ED) complaints

| Regression |  |  |  |
| --- | --- | --- | --- |
|  |  |  |  |
| Unadjusted models for each variable |  |  |  |
|  |  |  |  |
|  |  |  |  |
| **Characteristic** | **OR** | **95% CI** | **p-value** |
| Time interval ED arrival to ED departure in minutes bins |  |  |  |
| <4 Hours | — | — |  |
| 4-12 Hours | 1.21 | 0.91, 1.63 | .20 |
| >=12 Hours | 3.12 | 2.34, 4.18 | <.001 |
| Time interval ED arrival to seen by clinician in minutes bins |  |  |  |
| <30 Minutes | — | — |  |
| 30-60 Minutes | 1.24 | 0.94, 1.63 | .12 |
| 60-90 Minutes | 0.72 | 0.47, 1.09 | .13 |
| >=90 Minutes | 1 | 0.67, 1.45 | >.90 |
| Time interval ED arrival to roomed in minutes bins |  |  |  |
| <30 Minutes | — | — |  |
| 30-60 Minutes | 0.91 | 0.57, 1.41 | .70 |
| 60-90 Minutes | 0.73 | 0.34, 1.41 | .40 |
| >=90 Minutes | 0.73 | 0.40, 1.24 | .30 |
| Dispo admit | 0.72 | 0.57, 0.90 | .004 |
| Dispo discharge | 1.35 | 1.10, 1.67 | .005 |
| Boarding |  |  |  |
| <4 Hours | — | — |  |
| >=4 Hours | 1.4 | 0.95, 2.05 | .09 |
| ED hallway flag Yn |  |  |  |
| No | — | — |  |
| Yes | 1.01 | 0.81, 1.25 | >.90 |
| Visit from frequent ftilizer | 2 | 1.45, 2.73 | <.001 |
| Subsequent ED visit within 72 hours | 1.36 | 0.97, 1.88 | .07 |
| Arrival time of day |  |  |  |
| 7:00am-6:00pm | — | — |  |
| 7:00pm-6:00am | 1.25 | 1.01, 1.55 | .04 |
| Imaging | 0.43 | 0.35, 0.54 | <.001 |
| Proportion of hours during high volume 70 | 0.47 | 0.33, 0.67 | <.001 |
| Proportion of hours during high volume 80 | 0.4 | 0.26, 0.60 | <.001 |
| Proportion of hours during high volume 90 | 0.32 | 0.18, 0.55 | <.001 |
| Primary Insurance |  |  |  |
| Commercial | — | — |  |
| Medicaid | 1.2 | 0.90, 1.60 | .20 |
| Medicare | 1.14 | 0.87, 1.50 | .40 |
| Self-Pay | 1.5 | 0.85, 2.57 | .15 |
| Other/ Unknown | 1.14 | 0.36, 3.01 | .80 |

*OR,* odds ratio; *CI*, confidence interval
